# Light-Emitting Diode Photobiomodulation After Cerebral Ischemia

**DOI:** 10.3389/fneur.2019.00911

**Published:** 2019-08-22

**Authors:** Bárbara Argibay, Francisco Campos, María Perez-Mato, Alba Vieites-Prado, Clara Correa-Paz, Esteban López-Arias, Andrés Da Silva-Candal, Vicente Moreno, Carlos Montero, Tomás Sobrino, José Castillo, Ramón Iglesias-Rey

**Affiliations:** ^1^Clinical Neurosciences Research Laboratory, Health Research Institute of Santiago de Compostela (IDIS), Clinical University Hospital, Universidade de Santiago de Compostela, Santiago de Compostela, Spain; ^2^Optics Area, Department of Applied Physics, Faculty of Physics, Universitdade de Santiago de Compostela, Santiago de Compostela, Spain; ^3^Faculty of Optics and Optometry, Universitdade de Santiago de Compostela, Santiago de Compostela, Spain

**Keywords:** functional recovery, ischemic stroke, photobiomodulation therapy, magnetic resonance imaging, animal model, intracerebral hemorrhage

## Abstract

Photobiomodulation (PBM) therapy is a promising therapeutic approach for several pathologies, including stroke. The biological effects of PBM for the treatment of cerebral ischemia have previously been explored as a neuroprotective strategy using different light sources, wavelengths, and incident light powers. However, the capability of PBM as a novel alternative therapy to stimulate the recovery of the injured neuronal tissue after ischemic stroke has been poorly explored. The aim of this study was to investigate the low-level light irradiation therapy by using Light Emitting Diodes (LEDs) as potential therapeutic strategy for stroke. The LED photobiomodulation (continuous wave, 830 nm, 0.2–0.6 J/cm^2^) was firstly evaluated at different energy densities in C17.2 immortalized mouse neural progenitor cell lines, in order to observe if this treatment had any effect on cells, in terms of proliferation and viability. Then, the PBM-LED effect (continuous wave, 830 nm, 0.28 J/cm^2^ at brain cortex) on long-term recovery (12 weeks) was analyzed in ischemic animal model by means lesion reduction, behavioral deficits, and functional magnetic resonance imaging (fMRI). Analysis of cellular proliferation after PBM was significantly increased (1 mW) in all different exposure times used; however, this effect could not be replicated *in vivo* experimental conditions, as PBM did not show an infarct reduction or functional recovery. Despite the promising therapeutic effect described for PBM, further preclinical studies are necessary to optimize the therapeutic window of this novel therapy, in terms of the mechanism associated to neurorecovery and to reduce the risk of failure in futures clinical trials.

## Introduction

Photobiomodulation therapy has been investigated in the past few years as an alternative treatment for stroke and traumatic brain injury (TBI) in order to promote a neuroprotective effect and tissue regeneration (1–5). The main benefits attributed to brain PBM therapy are related to different biological processes such as increasing cerebral metabolic function, stimulating neurogenesis and synaptogenesis, and neuroprotection via anti-inflammatory, and antioxidant biological signaling (5, 6).

In the field of stroke, PBM applied in the acute phase has been suggested as a promising therapeutic approach for inducing functional recovery. Thus, some studies have demonstrated that 808–660 nm Low Level Laser Therapy (LLLT) applied after ischemia on experimental animals improved neurological rating scores without increasing body temperature, by direct illumination of the skin on shaved animals (7–10).

On the other hand, it has been shown that transcranial laser therapy within 24 h from stroke onset is a safe procedure, in terms of mortality or occurrence of adverse effects, when it is used on stroke patients (11, 12). LLLT used alone or combined with the thrombolytic treatment for stroke (recombinant tissue plasminogen activator) did not increase the risk of hemorrhagic transformation (13). Despite experimental evidences and human safety, a clinical trial designed to analyze the beneficial effect near-infrared laser therapy in stroke has showed negative results, in part because many of the parameters used (therapeutic time-window, laser intensity) were not sufficiently optimized for use in animal preclinical studies (14).

Light emitting diodes (LEDs) devices have emerged as an innovative source of brain PBM for a wide range of neurological conditions, and their use has been approved by the US Food and Drugs Administration. Different studies about LED-PBM have been mainly tested on cell *in vitro* assays (15–18), or in experimental animal studies like retinal alterations (19, 20), and its use in neuronal injures have been even tested in pathologies as TBI and stroke as a neuroprotectant approach, with contradictory results (5, 9, 21).

In this regard, the biological effects of PBM for the treatment of stroke have been widely explored using different light sources, wavelengths, and incident powers in preclinical studies. However, studies were mainly focused on the neuroprotective effects, while neurorecovery processes were evaluated only by functional tests or mortality rate, and the direct effect of PBM on recovery of the damage neuronal tissue was not accurately tested so far. In the field of magnetic resonance imaging (MRI), the interest in functional magnetic resonance imaging (fMRI) has been increasing significantly as a noninvasive tool to evaluate neural activity and the efficacy of neurorecovery therapies (22).

Therefore, to evaluate if LED-PBM could induce any positive effects of on neuronal recovery after stroke, in this study we have studied first the effect of PBM at different energy densities in cell culture, in terms of proliferation and viability. Secondly, we have studied the long-term recovery effect of LED-PBM in an animal model of transient ischemic stroke by means of reduction of ischemic lesion size, and finally we have tested for the first time functional recovery determined by fMRI in combination with functional behavioral tests.

## Materials and Methods

### Cellular *in vitro* Analysis of LED Photobiomodulation

It is well-known that neuronal growth and proliferation is one of the most relevant physiological mechanisms involved in the beneficial effects of light (1, 3). In this study, we have performed a previous *in vitro* evaluation about the effect of LED-PBM at different energy densities on cellular proliferation and viability.

For that purpose, C17.2 immortalized mouse neural progenitor cell line was used. C17.2 cells, provided by Prof. Mathias Hoehn (Max-Planck Institute, Cologne, Germany), were cultured in DMEM (78%), fetal bovine serum (10%), horse serum (5%), penicillin-streptomycin (1%) (Gibco Invitrogen, Paisley, UK), and amphotericin-B (1%) (Sigma-Aldrich, St. Louis, MO, USA).

To analyze the effect of LED-PBM on cells, an 830 nm LED array lamp (Quantum Spectralife 830 nm LED, Quantum Devices Inc., WI, USA) was used as light source. C17.2 cells were seeded (100,000 cells/well) in 6-well plates and placed at 15 cm from the LED source for PBM. Based on previous studies (15–17), light exposure was applied 2 consecutive days at 1, 5, and 10 mW during 6, 12, or 18 min, obtaining energy intensities from 0.2 to 6 J/cm^2^ (Supplementary Table 1). Non-exposed cells (control) were maintained under the same conditions as the 18 min exposed cells but without light stimulation.

To determine the influence of the PBM on the cellular proliferation, total cell count was performed with trypan blue staining (STEMCELL Technologies, Grenoble, France) and a Neubauer counting chamber (Blaubrand, Sigma-Aldrich, St. Louis, MO, USA) 24 h after the last PBM. Samples were diluted 1:5 with phosphate buffered saline (PBS) and 1:2 with Trypan blue. Cell count was performed by using an inverted microscope (Olympus IX51, Shinjuku, Tokyo, Japan).

For assessing the viability of the cells after PBM, supernatants were collected from well culture, including a negative control of lysed cells. Cell viability was determined by means of the lactate dehydrogenase assay (LDH) (Lactate Dehydrogenase Assay Kit. Sigma-Aldrich, St. Louis, MO, USA), following the manufacturer's protocol. In brief, supernatants were centrifuged at 1,000 rpm for 5 min and further incubated with LDH reagents for 20 min. Next, the plate was read in Synergy2 (Biotek Instruments, Vermont, USA) at 490 nm and the viability rate was calculated with respect to control and lysed values.

### Animals Studies

All experimental protocols involving the use of research animals have been approved by the University of Santiago de Compostela (Spain) ethics committee (ref: 15010/2019/004) and were performed according to the guidelines of the Animal Welfare Committee of the host institution and in accordance with applicable legislation of the European Union (86/609/EEC, 2003/65/EC, 2010/63/EU, RD 1201/2005 and RD 53/2013) and following the ARRIVE Guidelines for animal experiments.

Male Sprague–Dawley rats (Harlan Laboratories, Barcelona, Spain) weighing 300 ± 30 g (12 weeks old) were used in this study. Animals were kept in a controlled environment at 22 ± 1°C and 60 ± 5% humidity, with 12/12 h light/darkness cycles. Animals were fed *ad libitum* with standard diet pellets and tap water. All animals were housed individually.

All surgical procedures and MRI studies were conducted under sevoflurane (Abbott Laboratories, IL, USA) anesthesia (3–4%) using a carrier 65:35 gas mixture of N_2_O:O_2_. For fMRI experiments, animals were sedated with subcutaneous administration of medetomidine (Domitor®) (0.05 mg/kg bolus and 0.1 mg/kg/h infusion) during fMRI data acquisition. Respiration rate was continuously recorded non-invasively, and body temperature was kept constant by a feedback-controlled heating pad. After the experiment, sedation was antagonized with an intraperitoneal injection of atipamezole (0.1 mg/kg). No animal died during the follow-up period.

### Surgical Procedures for Transient Focal Ischemia

Transient middle cerebral artery occlusion (tMCAO) (60 min of occlusion) was induced by intraluminal occlusion of the middle cerebral artery with silicon handmade sutures (head diameter 300–350 μm), following the procedure described elsewhere (23, 24). A laser-Doppler flow probe (tip diameter 1 mm) attached to a flow meter (PeriFlux 5000; PerimedAB, Stockholm, Sweden) was placed on the thinned skull (area = 0.8 mm^2^, thickness = 0.2 mm) over the MCA territory (4 mm lateral to Bregma) to continuously record the relative cerebral blood flow (CBF) during the occlusion. Only animals with a reduction of CBF higher than 60%, and with effective reperfusion after retrieving the filament, were included in the study.

### Light Penetration Into the Rat Brain

Before testing the effect of LED-PBM on ischemic animal models, the penetration rate of light through different rat brain tissues (rodent skin, skull, and brain) was performed. For this purpose, the LED array lamp previously used in cellular studies was employed (830 nm, 10 mW/cm^2^). To determine the light penetration, a Newport 835 Laser Pico-Watt Digital Power Meter with 818 UV detector (Newport Corporation, Franklin, MA, USA) was used.

Light penetration through tissues was measured with isolated rat samples of skin, skull, and brain tissues. To evaluate the influence of the power in the penetration of light through different tissue samples, the LED lamp was located 15 cm away from the sample. To perform this analysis, a sample holder was made from black Plexiglas®, and consists of a base with an 8 mm hole in its center where we placed the samples and 3 black Plexiglas® walls. Two photodetectors let the simultaneous measurement of light power transmitted through the different tissues we placed on the holder, and a portion of the incident light.

Light penetration was calculated and performed 3 times (*n* = 2 animals). Data were processed using a program designed for that purpose in C^++^ (Borland, Texas, USA). First, we measured the power without any sample between the light source and the detector (light in mW/cm^2^ across of air) and then we calculated the percentage of transmitted light when samples were placed on the system (light in mW/cm^2^ across of tissue). Relative penetration percentage values were calculated using the following formula obtained from a previous study (25):

(1) $$\begin{array}{l} 100\;\%\;X\frac{\left(Light\;across\;tissue\;\left[\frac{mW}{cm^{2}}\right]\right)}{\left(Light\;\left[\frac{mW}{cm^{2}}\right]\right)} \end{array}$$

### *In vivo* LED Photobiomodulation Neurorecovery Analysis

Because TBI represents the type of neurological damage closest to ischemic injury, we used similar continues LED PBM protocol in our ischemic animal model (5). In our case, in order to reduce as much as possible the stressful effects that LED-PBM could induce on the experimental animals and interfere in the fMRI experiments (26), we decided to test two different continuous LED-PBM protocols; Group 1: 30 min of light treatment, 1 day/week during 12 weeks (*n* = 6), and Group 2: 30 min of light treatment, 3 days/week during 12 weeks (*n* = 6). An additional ischemic group without treatment was used as control Group (*n* = 6). Animals were randomly assigned to the three experimental groups. LED-PBM was initiated 24 h after the onset of ischemia in awake and freely moving conditions. Head animals were shaved before light stimulation and the light exposure was performed individually.

Based on the cellular *in vitro* experiments and light penetration analysis on cerebral tissues, LED-PBM was induced with a power density of 10 mW/cm^2^ to 1 cm^2^ of area (at 15 cm of the source) with a light intensity of 18 J/cm^2^.

### Magnetic Resonance Imaging

Magnetic Resonance Imaging and fMRI studies were conducted on a 9.4 T horizontal bore magnet (BrukerBioSpin, Ettlingen, Germany) with 20 cm wide actively shielded gradient coils (440 mT/m). MR images were acquired using a cross-coil setup, consisting of a linear birdcage resonator of 7 cm diameter, for transmission, and a 2 × 2 arrayed surface coil for signal detection.

Infarct volume was determined by means of T2-weighted images acquired at 24 h (defined as 0) and 1, 3, 5, 7, and 12 weeks after the onset of ischemia using a multi-slice multi-spin-echo (MSME) sequence with the following acquisition parameters: repetition time (TR) = 3 s, 16 echoes with echo time (TE) = 9 ms, 75 KHz spectral bandwidth, flip angle (FA) = 180°, 14 slices of 1 mm thickness, field of view (FOV) of 19.2 × 19.2 mm^2^ (with saturation bands to suppress signal outside this FOV), a matrix size of 192 × 192 (isotropic in-plane resolution of 0.1 mm/pixel), and implemented without fat suppression option.

All images were processed using the ImageJ software (Rasband, W.S., ImageJ, NIH) on an independent computer workstation. Infarct volumes were quantified from T2-weighted images averaged all echoes by a researcher blinded to the treatment groups.

### BOLD fMRI Methodology

Functional MRI was achieved using BOLD contrast, starting at least 1 h after the induction of the sedation. fMRI experiments were performed 1 week before tMCAO and 0 (24 h), 1, 3, 5, 7, and 12 weeks after ischemia, acquiring sets of 115 consecutive spin-echo Echo-Planar-Images (SE-EPI) with TE/TR = 30/3,000 ms, 5 slices of 2 mm thickness, FOV of 19.2 × 19.2 mm^2^ covered by a 64 × 64 matrix, i.e., an in-plane resolution of 0.3 mm/pixel. Home-built steel needle (30 G) electrodes were placed subcutaneously into both forepaws. Alternating unilateral forepaw stimulation was performed using rectangular pulses (2 mA, 8 Hz, 0.3 ms, STG2004 stimulator from Multi Channel Systems, Germany) following a paradigm in which a block, composed by a 45 s resting period and 15 s activation period, was repeated five times, ending with an additional 45 s resting period for a total experimental time of 5 min 45 s (5 × [45 OFF + 15 ON] + 45 OFF). BOLD fMRI technique is based on the differential magnetic properties of oxygenated (diamagnetic) and deoxygenated (paramagnetic) hemoglobin. Upon neural activation, changes in local CBF, cerebral blood volume (CBV), and cerebral metabolic rate of oxygen consumption (CMRO2) leads to a locally increased ratio of oxygenated over deoxygenated hemoglobin, resulting in an enhancement in T2^*^-weighted signal intensity (27).

BOLD fMRI was conducted, alternating three times between each hemisphere, and animals were allowed to rest for 10 min between stimulation sessions. After pre-processing, the BOLD signal was modeled as the convolution of the applied stimulation design (block or event-related design) with the hemodynamic response function (HRF) in order to have a better estimation of the true design-related BOLD signal. Finally, statistics was performed on the model estimates defining brain regions activated (27).

Statistical parametric activation maps were constructed with the software STIMULATE (28). The time course of each pixel during forepaw stimulation was examined using a paired Student's *t*-test (*p* < 0.05) according to previous studies (29, 30), comparing the mean baseline signal value with the mean of the stimulated signal value. Only clusters that included at least four adjacent activated pixels were considered as positive activation areas.

### Sensorimotor Test: Cylinder Test

A cylinder test (31) was used to evaluate the functional deficit and was performed 1 week prior to tMCAO (to assess the basal locomotor symmetry of the animals) and 24 h (defined as 0), 1, 3, 5, 7, and 12 weeks after ischemia during the darkness cycle (Supplementary Material).

### Brain Histological Analysis

Animals were sacrificed 12 weeks after surgical treatment, and brain tissue was processed for histological analysis, following the procedure described elsewhere (32). Neuronal nuclear protein (Fox3) and glial fibrillary acidic protein (GFAP) (Sigma-Aldrich) labeling were combined with DAPI stain (Thermo Fisher Scientific, Waltham, MA, USA). In addition, neurogenesis was examined in the striatal region by immunolabeling a proliferating cell marker (Ki-67, Abcam) and doublecortin (DCX; an immature progenitor cell marker) (Dako, Barcelona, Spain). Doblecortin and Ki-67 positive, GFAP-positive and Fox3-positive cells and nuclei were counted manually in ImageJ software (Supplementary Material).

### Statistical Analysis

All data are presented as the mean and standard deviation of the mean (mean ± SD). The Kolmogorov-Smirnov test was performed in order to check the normality of distributions. Two-way analysis of variance (ANOVA) followed by *post-hoc* Bonferroni evaluation was used for multiple groups to determine significant differences. Statistical significance was set at *p* < 0.05. The statistical analysis was conducted using IBM SPSS Statistics for Macintosh, Version 18.0. (SPSS Inc., IL, USA). Statistical analysis was performed by a blinded researcher to the treatment. Sample size was based on expected variances: α = 0.05 (confidence level: 95%) and β = 0.20 (power: 80%).

To achieve a power of 80% to detect differences in the contrast of the hypothesis null H0 (LED-PBM treatment could reduce the ischemic lesion by 30%) taking into account that the level of significance is 5%, the number of animal in each group is *n* = 6. The calculation has been made with the program Ene 3.0.

## Results

### *In vitro* Analysis of Photobiomodulation

**Figure 1:**
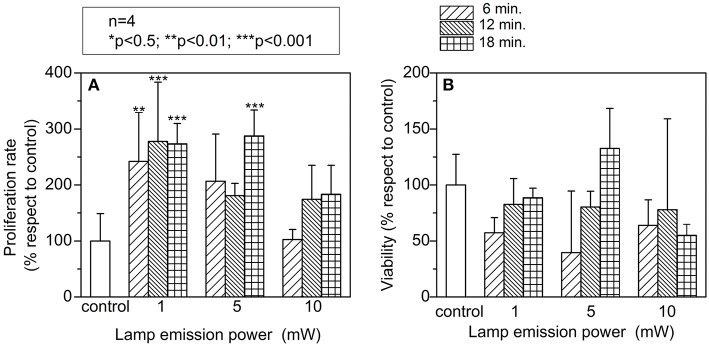
*In vitro* study to evaluate: **(A)** cellular proliferation; cell count was found significant increased at low emission powers and exposures times. **(B)** Viability; photostimulaltion did not produce toxic side effects *in vitro*. A two-way analysis of variance (ANOVA) followed by *post-hoc* Bonferroni evaluation was used. (*n* = 4; *p < 0.5; **p < 0.01; ***p < 0.001). Lamp emission power at three different exposure time (6, 12, and 18 min).

Analysis of cellular proliferation after PBM was significantly increased with a density power of 1 mW in all different exposure times tested, however higher intensities did not show higher effects (Figure 1A). To determine if the PBM was associated with cellular death, LHD assay was performed (Figure 1B). Although it did not achieve a significant difference, a higher cell death was observed only in those cells treated for 18 min with 5 mW of light exposure. Based on these *in vitro* results, we established 0.2–0.6 J/cm^2^ (Supplementary Table 1) as the optimal light energy intensity needed to induce any beneficial effect on the neuronal tissue in the *in vivo* animal study.

### *In vivo* Photobiomodulation Effect on Functional Recovery and Ischemic Lesion

A total number of 20 animals were used in the study: 2 animals involved in the light penetration analysis into the brain, and 18 animals in the neurorecovery analysis. Two animals died during the surgery, and 3 animals did not meet the inclusion criteria in relation to the reduction of CBF.

Light penetration was determined through the different tissues, resulting 16 ± 3% through the shaved skin, 10 ± 3% through the skull, 0.9 ± 0.4% through right brain hemisphere (ischemic), and 0.9 ± 0.4% through left brain hemisphere (healthy).

Analysis of light penetration study through different biological tissues from rat (rodent skin, skull, and brain) showed that the decay of light intensity rate along the deep brain tissue resulted about 2.5 × 10^−2^ J/cm^2^. Based on this analysis, light PBM intensity was adjusted to 18 J/cm^2^ that allowed inducing on brain cortex with a light intensity of 0.28 J/cm^2^, close to that values established in the previous *in vitro* study (0.2–0.6 J/cm^2^).

**Figure 2:**
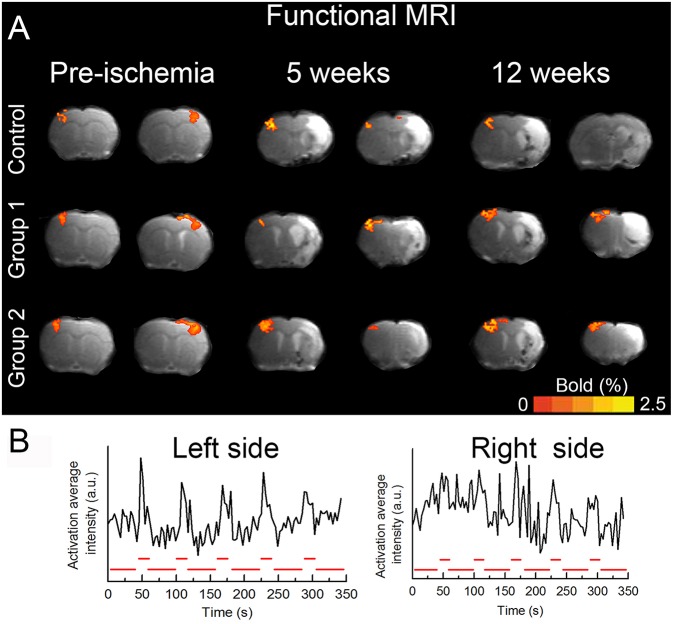
Functional brain recovery observation. **(A)** Representative BOLD images obtained during pre-cerebral ischemia, 5 and 12 weeks after the insult in both hemispheres. **(B)** Alternating unilateral forepaw stimulation was performed using rectangular pulses. BOLD fMRI was conducted alternating three times between each hemisphere, and animals were allowed to rest for 10 min between stimulation sessions. The time course of each pixel during forepaw stimulation was examined using a paired Student's *t*-test (*p* < 0.05). Only clusters that included at least four adjacent activated pixels were considered as positive activation areas. (*n* = 6 animals/group). All animals exhibited BOLD signal in both hemispheres (measured separately) prior to the cerebral ischemia. The animals were followed for 12 weeks and all of them exhibited BOLD signal in the contralateral side but none of them showed BOLD activation in the ipsilateral side.

**Figure 3:**
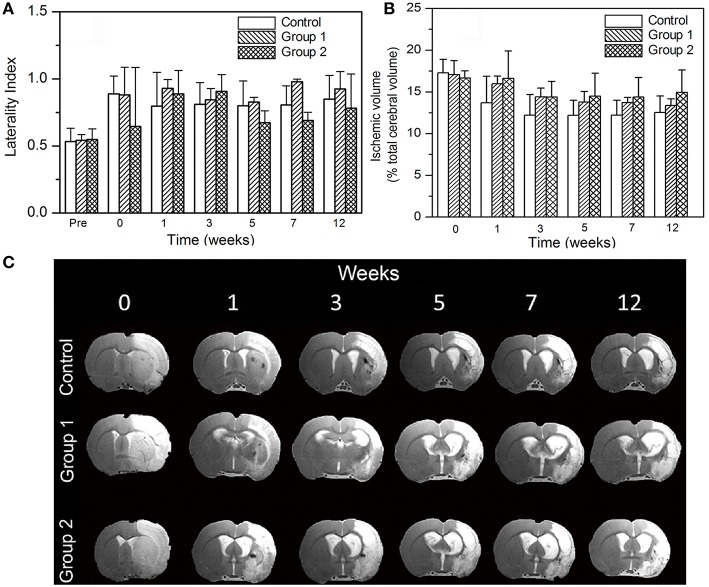
*In vivo* study: **(A)** Cylinder test results obtained from the three studied groups. **(B)** Ischemic volume results of the three groups; No significant differences were observed between groups up to 12 weeks. **(C)** T2-weighted MR images of a representative brain of each photostimulated group.

Functional MRI was performed in all animals prior to the tMCAo and all of them exhibited bilateral BOLD signal (Figure 2). Nevertheless, 12 weeks after PBM treatment, animals did not show the recovery BOLD signal in the ischemic hemisphere with none of the protocols used. In line with fMRI results, analysis of the profile of lateral index (that determinates the forelimb asymmetry) analyzed during the period of follow up, did not show a significant improvement in the functional deficit in the treated groups respect to the control group (Figure 3A). Nonetheless, the treated groups showed a reduction of the use of the impaired forelimb, but the difference did not reach statistical significance compared to the control group.

Figure 3B detailed the evolution of the lesion sizes of ischemic animals. All the animals included in the study presented a basal ischemic lesion (<18%) determined by MRI. According to our experimental design, a reduction of 30% in the ischemic lesion was not found. In line with this, no significant differences in volume reduction were observed for the groups compared to control. Under these experimental conditions, analysis of infarct volume showed that LED photostimulation doesn't reduce the ischemic lesion in the treated animals compared with the control group (Figure 3C). Besides, subarachnoid hemorrhages were found in all groups (control group included) due to the large ischemic lesions induced with the tMCAO model.

**Figure 4:**
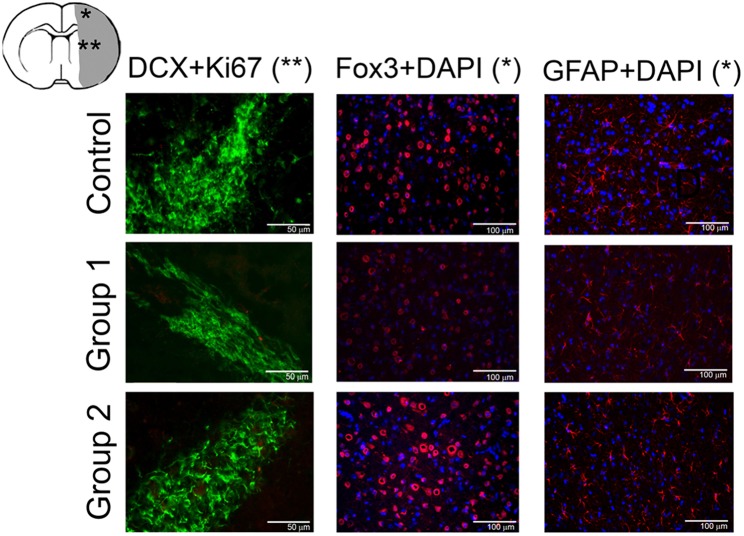
Histological analysis of neurons (Fox3), astrocytes (GFAP), and neurogenesis (DCX and Ki 67) of animals from each experimental group at 12 weeks after ischemic lesion. Cortical (*) and subcortical brain regions (**).

Histological analysis of a neuronal marker (Fox3), glial marker (GFAP), and neurogenesis performed 12 weeks after stroke onset did not reveal expression differences in either immunolabeling (Figure 4). All animals used were included in the study and no mortality associated to PBM was observed.

## Discussion

In the present study, PBM with 830 nm LED at low energy intensity (0.28 J/cm^2^) did not reduce the infarct volume or improve the functional recovery 12 weeks after the onset of the ischemia. The electrical activity and therefore fMRI signal in response to the forepaw stimulation was observed exclusively in the contralateral hemisphere, evidencing no functional recovery after cerebral ischemia in the ipsilateral side. Other therapeutic studies with very different treatments, as stem cells, but where fMRI was used to evaluate the functional recovery after ischemic stroke have evidenced that the regaining of BOLD signal was produced between the 7th and the 10th week after the administration (33). Therefore, and although the therapy is completely different, we coinsured that 12 weeks were a prudential period to see some effect of LED-PBM through fMRI.

Numerous studies have found positive effects conferred by PBM from decreasing inflammation, alleviating oxidative stress, and improving mitochondrial function (5–10). Some studies have evidenced reduction of cerebral ischemic lesions, better functional outcome or cell neurogenesis in different animal models (8–10). However, in line with our results, these PBM therapies were mainly applied within the first 6 h from stroke onset, while no benefit was observed at longer time-points. Similarly, the clinical Phase III (NEST-3) study about the therapeutic effect of PBM that did not find significant benefit in stroke patients (14).

In our study, 24 h after stroke was selected as the time-point to start the LED-PBM treatment, in order to study repair-based therapies complement acute therapies (intravenous or intraarterial fibrinolysis, thrombectomy). Besides, within the first 24 h after stroke, patient requires clinical care, MRI, or Computed Tomography (CT) studies, which would limit the possibility of implementing the PBM protocols.

We have evaluated the evolution of the ischemic brain by using MRI and fMRI after *in vivo* PBM, however our study also shows some weaknesses. First, the use of an ischemic cell model [oxygen-glucose deprivation (OGD)] would help understand basic biochemical and cellular mechanisms involved in the effects of light. Second, it would be possible that prolonged individual housing of animal prevented optimal recovery and negatively impacted to detect a therapeutic effect. Third, a deep histological analysis would contribute to elucidate the light effects in the damaged brain at a cellular level, or even to describe possible endogenous angiogenic or neurogenic processes stimulated by the LED light. In addition, in this study we have used a tMCAO model that induces large ischemic lesion with a high sensorimotor cortex affectation, that can limit the functional recovery as it has been reported in other studies (33), and potentially explain the lack of PBM effect observed in ours animals. Indeed, this could also reflect that this treatment could be more appropriate for smaller stroke lesions.

Hundreds of clinical trials testing protective agents have failed despite efficacy in experimental models. In this line, from a long time ago, negative and neutral studies have been required to help move novel treatments to human and reduce the risk of failure in futures clinical trials (34, 35). Therefore, these new findings try to contribute to optimize the potential use PBM as novel and non-invasive therapy for ischemic stroke before moving to clinical studies.

## Conclusion

Under our experimental conditions, we have not observed a beneficial effect of LED photobiomodulation applied 24 h after the onset of ischemia in animal models, mainly in terms of neuronal recovery determined by fMRI. Further preclinical studies in this field are necessary in order to optimize the therapeutic window of this novel therapy, the mechanism associated and to reduce the risk of failure in futures clinical trials.

## Data Availability

All datasets generated for this study are included in the manuscript/Supplementary Files.

## Author Contributions

BA, RI-R, and MP-M performed the MRI scanning and fMRI and preparation of manuscript. AV-P, AD, CC-P, and EL-A contributed in the animal surgeries and behavior analysis and preparation of manuscript. VM and CM designed light experiments and analysis and contributed to the preparation of the manuscript. BA, JC, TS, and FC contributed to the execution of experiments, data analysis, preparation of manuscript, and experimental design. All authors have read the manuscript, agree that the work is ready for submission to a journal, and accept responsibility for the manuscript's content.

### Conflict of Interest Statement

The authors declare that the research was conducted in the absence of any commercial or financial relationships that could be construed as a potential conflict of interest.
