# Supplementary material for: Light-Emitting Diode Photobiomodulation After Cerebral Ischemia
Source: Front Neurol. 2019 Aug 22;10:911. doi: 10.3389/fneur.2019.00911 (PMC6713875; doi:10.3389/fneur.2019.00911)

**Supplementary Material**

MATERIAL AND METHODS

**1. Supplementary Table 1:** 830 nm LED array lamp parameters for *in vitro* photobiomodulation.

| Power  (mW) | Exposure Time  (minutes) | Energy Intensity  (J/cm^2^) |
| --- | --- | --- |
| 1 | 6 | 0.2 |
|  | 12 | 0.4 |
|  | 18 | 0.6 |
| 5 | 6 | 1 |
|  | 12 | 2 |
|  | 18 | 3 |
| 10 | 6 | 2 |
|  | 12 | 4 |
|  | 18 | 6 |

**2. Sensorimotor test: Cylinder test**

A cylinder test was used to evaluate the functional deficit and was performed 1 week prior to tMCAO (to assess the basal locomotor symmetry of the animals) and 24 hours (defined as 0), 1, 3, 5, 7 and 12 weeks after ischemia during the darkness cycle.

Functional outcome was evaluated using the cylinder test. Exploratory behaviour in each animal was recorded for 10 min in a 20 × 30 cm cylinder in a darkened room with an infrared video camera (Sony, Tokyo, Japan). All functional assessments were conducted during the animals’ active periods (the first 6 h of the dark period). Scores were obtained from a total number of 10 full rears to control for differences in rearing between animals. Forelimb asymmetry was calculated using the formula: 100 × (ipsilateral forelimb use + 1/2 bilateral forelimb use)/total forelimb use observations.

**3. Brain histological analysis**

Animals were sacrificed 12 weeks after ischemic injury Animals were deeply anaesthetised with sevoflurane (6% in a mixture of 70% NO2 and 30% O2) and transcardially perfused with 100 mL of PBS (pH 7.4) followed by 150 mL of 4% (v/v) formaldehyde.

Brains were carefully removed from the skull and sectioned at 2 mm thick using a matrix. Slices were post-fixed by immersion in 4% (v/v) formaldehyde overnight, washed in PBS, and cryoprotected in a solution of 30% (v/v) sucrose in PBS with 0.05% sodium azide. Slices were embedded in OCT compound (Sakura Finetek, Torrance, CA, USA), flash-frozen with liquid nitrogen, and cut into 8 μm-thick slices using a cryostat (Sakura Finetek).

Neuronal nuclear protein (Fox3, Sigma-Aldrich) and astrocyte marker glial fibrillary acidic protein (GFAP, Sigma Aldrich) labeling was combined with DAPI stain (Thermo Fisher Scientific, Waltham, MA, USA). In addition, neurogenesis was examined in the striatal region by immunolabelling with a proliferating cell marker (Ki-67, Abcam) and doublecortin (DCX; Dako).

Slices were incubated overnight at RT with primary antibodies diluted in PBS with 0.2% (v/v) Triton X-100 and 15% (v/v) normal serum derived from the same species as the secondary antibody. The dilutions used for the primary antibodies were those specified in the data sheet. Slices were then washed with PBS and incubated for 1h at RT in the dark with the following secondary antibodies: biotinylated horse anti-rabbit (BA-1100; Vector Laboratories), biotinylated horse anti-mouse (BA-2001; Vector Laboratories, Peterborough, UK), DyLight 488 horse anti-mouse (IGGDY488H-OIMG-CUSTOM; Immunostep), or DyLight 488 goat anti-rabbit (DI-1488; Vector Laboratories. The secondary antibodies were diluted 1:200 in PBS with 0.2% (v/v) Triton X-100. Slices were washed and incubated for 30 min in the darkness with DyLight 594 streptavidin (SA-5594; Vector Laboratories) diluted 1:500 in PBS with 0.2% (v/v) Triton X-100. Finally, the slices were washed and incubated with DAPI stain diluted 1:6,000 in PBS for 10 min in the dark.

Slices were mounted with Aqua-Poly/Mount (Polysciences, Warrington, PA, USA) and photographed using a Leica DMI6000 B microscope with Leica Application Suite Advanced Fluorescence software, version 1.0.0 (LAS AF; Leica). A quantitative analysis of Doblecortin and Ki-67, GFAP and Fox3 immunoreactivity was performed using three animals from each group. Photomicrographs were obtained from the striatum and cortex in ischemic hemispheres. Replicate photomicrographs were obtained from two consecutive slices containing the central part of the injured region (between 0.7 mm anterior and 0.3 mm posterior to the bregma). Doblecortin and Ki-67 positive, GFAP-positive and Fox3-positive cells and nuclei were counted manually in ImageJ software.


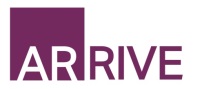
The ARRIVE Guidelines Checklist

Animal Research: Reporting In Vivo Experiments

Carol Kilkenny^1^, William J Browne^2^, Innes C Cuthill^3^, Michael Emerson^4^ and Douglas G Altman^5^

*^1^The National Centre for the Replacement, Refinement and Reduction of Animals in Research, London, UK, ^2^School of Veterinary Science, University of Bristol, Bristol, UK, ^3^School of Biological Sciences, University of Bristol, Bristol, UK, ^4^National Heart and Lung Institute, Imperial College London, UK, ^5^Centre for Statistics in Medicine, University of Oxford, Oxford, UK.*

|  | | ITEM | RECOMMENDATION | Section/ Paragraph | |  |
| --- | --- | --- | --- | --- | --- | --- |
| 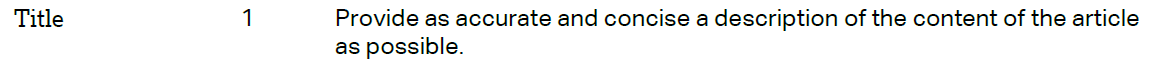 | | | Title | |  |  |
| 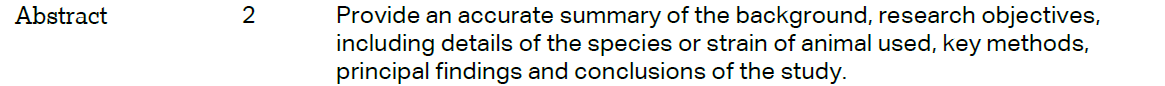 | | | Abstract | |  |  |
| INTRODUCTION | | |  | |  |  |
| 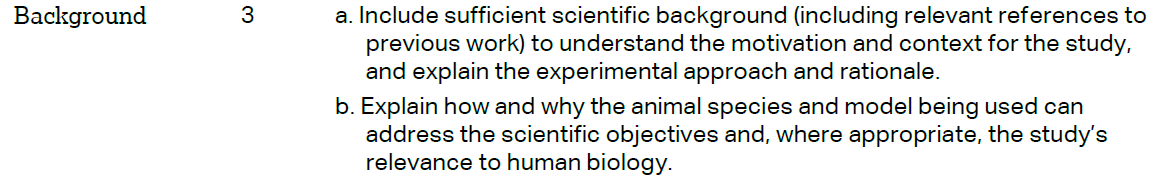 | | | Introduction  Introduction | |  |  |
| 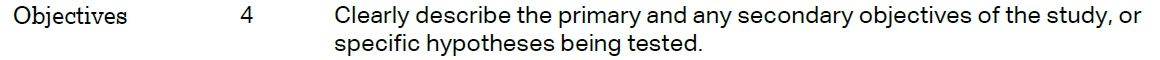 | | | Introduction | |  |  |
| METHODS | | |  | |  |  |
| 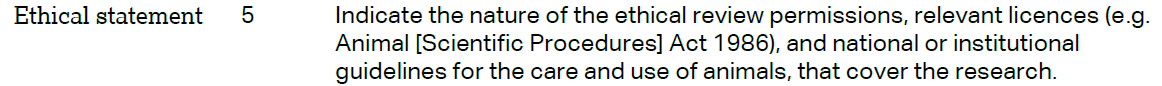 | | | Animal studies section | |  |  |
| 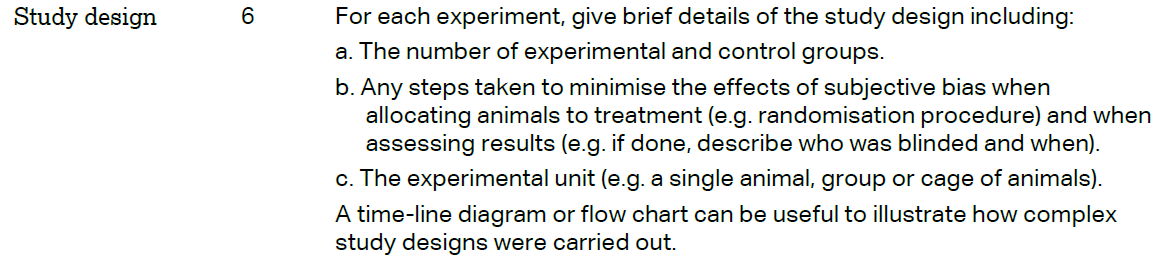 | | | Material and Methods sections | |  |  |
| 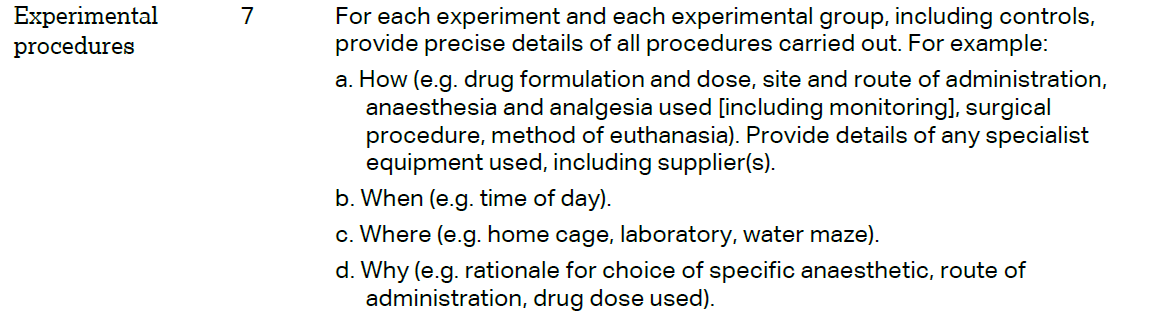 | | | Material and Methods sections | |  |  |
| 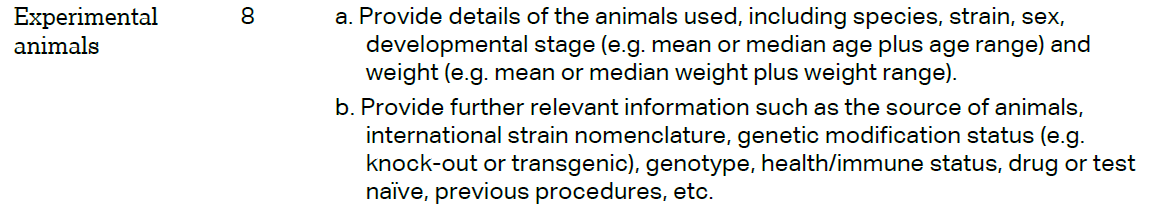 | | | Material and Methods sections | |  |  |
| 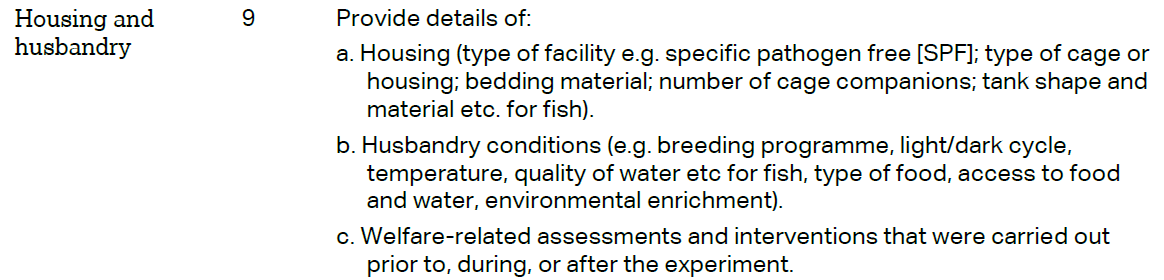 | | | | Animal studies section | | |
| 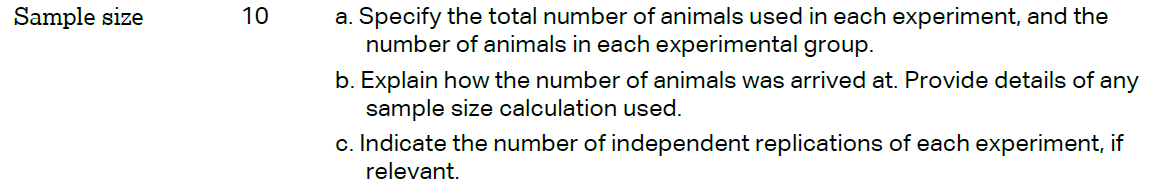 | | | | Animal studies section  Light penetration and In vivo Led sections | | |
| 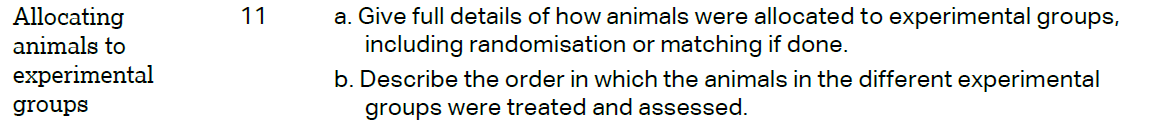 | | | | Material and Methods sections | | |
| 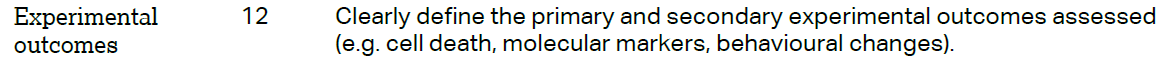 | | | | Material and Methods sections | | |
| 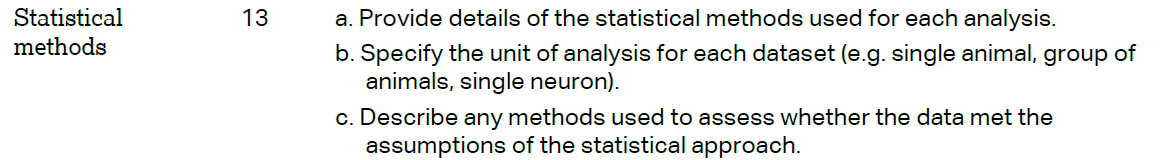 | | | | Statistical section | | |
| RESULTS | | | |  | | |
| 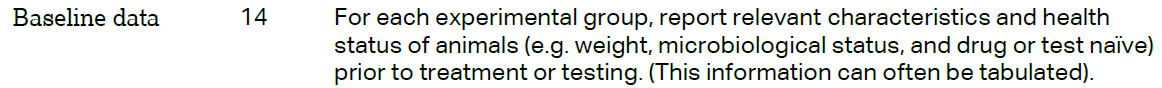 | | | | Results sections  Figures1-4 | | |
| 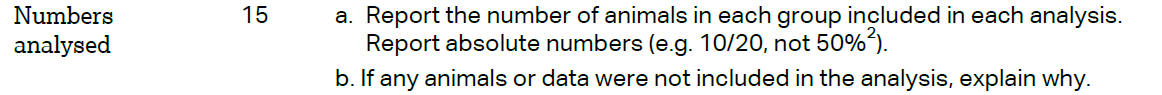 | | | | Material and Methods sections | | |
| 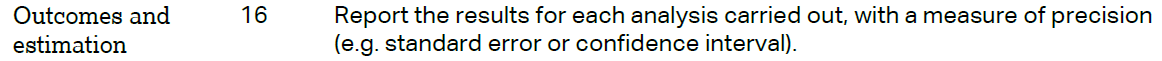 | | | | Results sections | | |
| 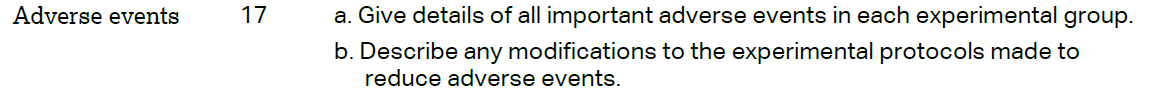 | | | | Results sections | | |
| DISCUSSION | | | |  | | |
| 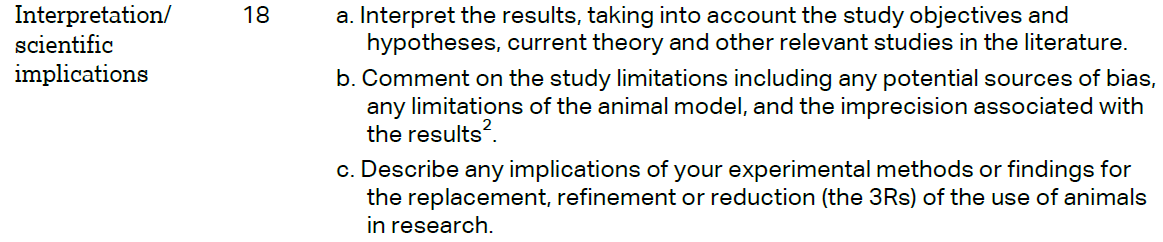 | | | | Discussion section | | |
| 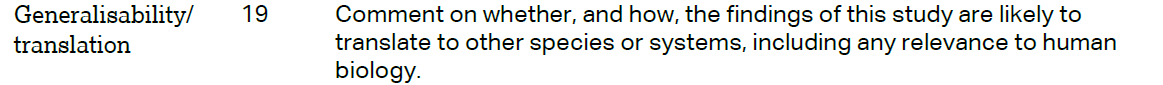 | | | | Discussion section | | |
| 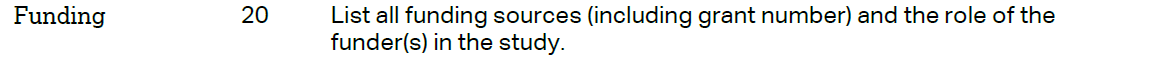 | | | | | Funding section | |


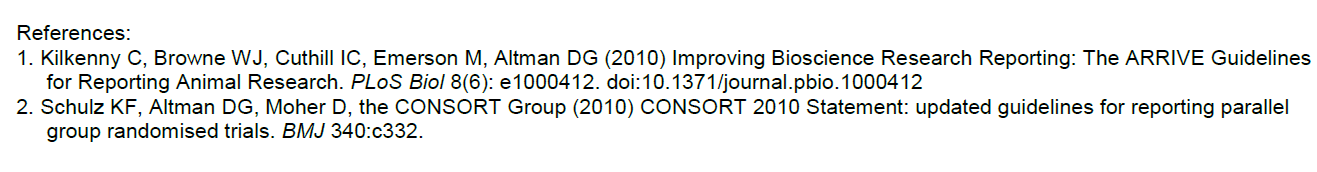

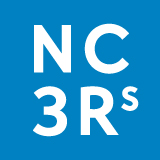

Supplement: Supplementary file 1 [file Data_Sheet_1.docx]
